# Cardiac implantable electronic devices in the setting of tricuspid valve intervention: Risks, options, extraction and future perspectives: A state-of-the-art review

**DOI:** 10.1016/j.hroo.2025.02.016

**Published:** 2025-02-28

**Authors:** Alphonsus Liew, Nadeev Wijesuriya, Sandra Howell, Felicity de Vere, Joshua Wilcox, Tiffany Patterson, Steven Niederer, Christopher Aldo Rinaldi

**Affiliations:** 1 School of Biomedical Engineering and Imaging Sciences, King’s College London, London, United Kingdom, Department of Cardiology, Guy’s and St Thomas’ NHS Foundation Trust, London, United Kingdom; 2 Cardiovascular, Faculty of Life Sciences & Medicine, King’s College London, United Kingdom, Cardiovascular Department, Guy’s & St. Thomas’ NHS Foundation Trust, United Kingdom; 3 National Heart and Lung Institute, Imperial College London, United Kingdom

**Keywords:** Tricuspid valve replacement, Tricuspid valve repair, Lead jailing, Lead entrapment, Tricuspid regurgitation, Coronary sinus pacing, Tricuspid valve-in-valve

## Abstract

Tricuspid regurgitation is associated with increased risk of heart failure and mortality. To address this, tricuspid valve (TV) interventions in the form of transcatheter and surgical TV procedures have rapidly emerged. TV interventions are associated with a significant risk of conduction system disease necessitating permanent pacemaker (PPM) implantation. Limited data and current guidelines suggest that transvalvular PPM should be avoided to preserve the function of replaced or repaired TVs. With the rapid increase of transcatheter tricuspid valve-in-valve replacements in the last decade, the incidence of pre-existing PPM lead entrapment poses a clinical challenge in which lead extraction is indicated, particularly in the context of device infection. In this review article, we discuss the risks of conduction system disease associated with different TV interventions, the options for pacing, defibrillator therapy and cardiac resynchronization therapy that spare the TV, and the management of pre-existing transvalvular pacing leads before TV intervention. Furthermore, we discuss the future perspectives in this field, including a completely leadless cardiac resynchronization therapy-defibrillator system, and propose an algorithm for cardiac implantable electronic devices (CIED) implantation during or after TV intervention.

## Graphical abstract

**Figure:**
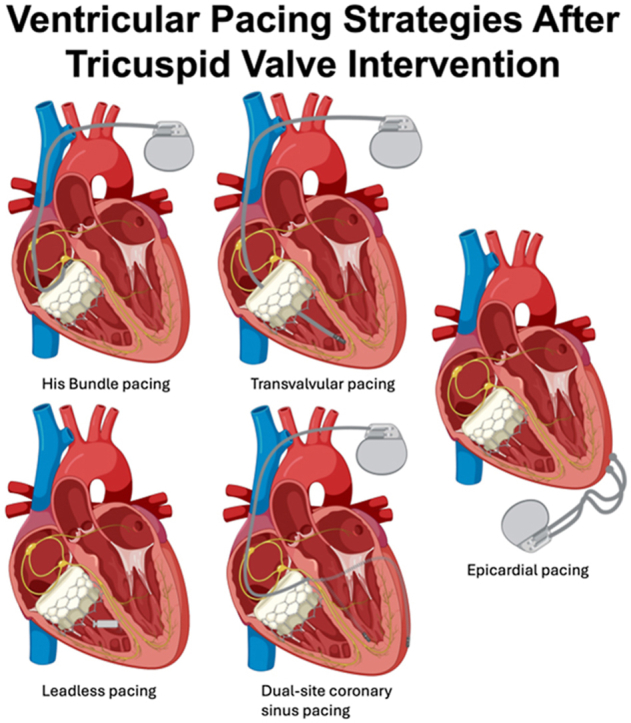

**Key Findings**

▪ Tricuspid valve replacement and repair are associated with significant risk of conduction system disease requiring PPM implantation, with a risk of 8%–35% and 3%–14%, respectively.
▪ Transvalvular pacing after TV intervention should be avoided.
▪ The options for pacing after TV intervention that avoids valve interaction include His bundle pacing, coronary sinus pacing, epicardial pacing, and leadless pacing.
▪ Where there is an indication for defibrillator therapy after TV intervention, the use of S-ICD or EV-ICD is preferred. Where there is a concurrent need for resynchronization therapy, the combination of S-ICD with DS-CS or DS-CS alone with an ICD lead in 1 of the CS branches may be considered, with the caveat that the latter option is largely evidence free.
▪ Lead extraction of leads jailed by TVR is technically very challenging and high risk. Potential complications include significant paravalvular leak, prosthesis or vegetation embolization, damage to the TV annulus from wide-caliber extraction sheaths, or snapping of embedded leads.
▪ The jailing of pacemaker leads during TVR should be avoided unless the risk of lead extraction is deemed to be very high.
▪ A completely leadless CRT-D system may be an option in the near future after TV intervention.

## Introduction

Tricuspid regurgitation (TR) is associated with increased risk of heart failure and mortality.^1^ Indeed, 1-year mortality of severe TR with medical therapy alone is between 36% and 42%, irrespective of cause.^2^^,^^3^ Recognizing the negative impact of tricuspid valve (TV) and right ventricular dysfunction on mortality, current guidelines recommend TV surgery (TVS) in those with severe primary or secondary TR undergoing left-sided heart surgery.^4^^,^^5^ It is therefore unsurprising that the rates of TVS and transcatheter TV intervention (TTVI), including repair and replacement, have been rising.^6^^,^^7^ In line with this rise is the increasing adoption transcatheter tricuspid valve-in-valve replacement (TTVIV) for the treatment of failing TV bioprosthesis, particularly in patients with previous open-heart surgeries who are deemed to be too high risk for further open-heart surgery.

TVS and TTVI are associated with increased risk of conduction system disease, often necessitating the implantation of a permanent pacemaker system (PPM) perioperatively. The best strategy for permanent pacing in this context is unclear, because a transvalvular approach may affect the function of the replaced or repaired valve, culminating in recurrence of significant TR, and epicardial pacing is associated with poor durability. Another issue that will become more prevalent is the management of existing transvalvular pacemaker leads at the time of TVS or TTVI. With the rise of valve-in-valve procedures, it is expected that a rise in “jailed” or entrapped leads will be observed.

In this review, we discuss the risk of PPM implantation after different forms of transcatheter and surgical tricuspid valve intervention, the different strategies for delivering permanent pacing or defibrillator and cardiac resynchronization therapy (CRT) in this context, and lead extraction of pacing leads jailed by tricuspid valve replacements (TVR). Additionally, we compare the management options of pre-existing transvalvular leads before TVS or TTVI, namely, extraction and reimplantation after tricuspid valve intervention or entrapment of the leads during TVS or TVI.

## Risk of significant conduction system disease after TVS or TTVI

### Tricuspid valve surgery

Current American and European guidelines recommend concomitant TVS in patients already undergoing mitral valve surgery.^4^^,^^5^ Both tricuspid valve replacement (TVR) and tricuspid valve repair (TVr) have been associated with an increased risk of conduction system disease requiring PPM implantation, with TVR carrying a 3-fold risk of PPM implantation compared with TVr.^6^^,^^8^^,^^9^

A retrospective study of 13,294 patients who had undergone TVS found that 18.9% of patients required PPM within 30 days of discharge from index admission.^8^ TVR had a higher risk of PPM implantation compared with TVr (relative risk [RR], 3.2; confidence interval [CI], 2.16–4.75; *P* < .0001).^8^ The risk was further increased in those who had undergone isolated TVS, with 27.3% of TVR and 12.6% of TVr requiring PPM implantation. Another retrospective analysis involving 5005 patients who had undergone isolated TVS found a similar rate of PPM implantation of 26% (TVR 35% and TVr 13.4%).^6^ Similarly, a randomized controlled trial comparing combined mitral valve surgery plus tricuspid annuloplasty compared with mitral valve surgery alone found the incidence of postoperative PPM requirement to be 14.1% after TVS.^9^ Other risk factors for postoperative PPM include female sex, cross clamp time of >60 minutes, and preoperative conduction defects such as underlying bundle branch block. ^8^^,^^10^, ^11^, ^12^

**Figure 1:**
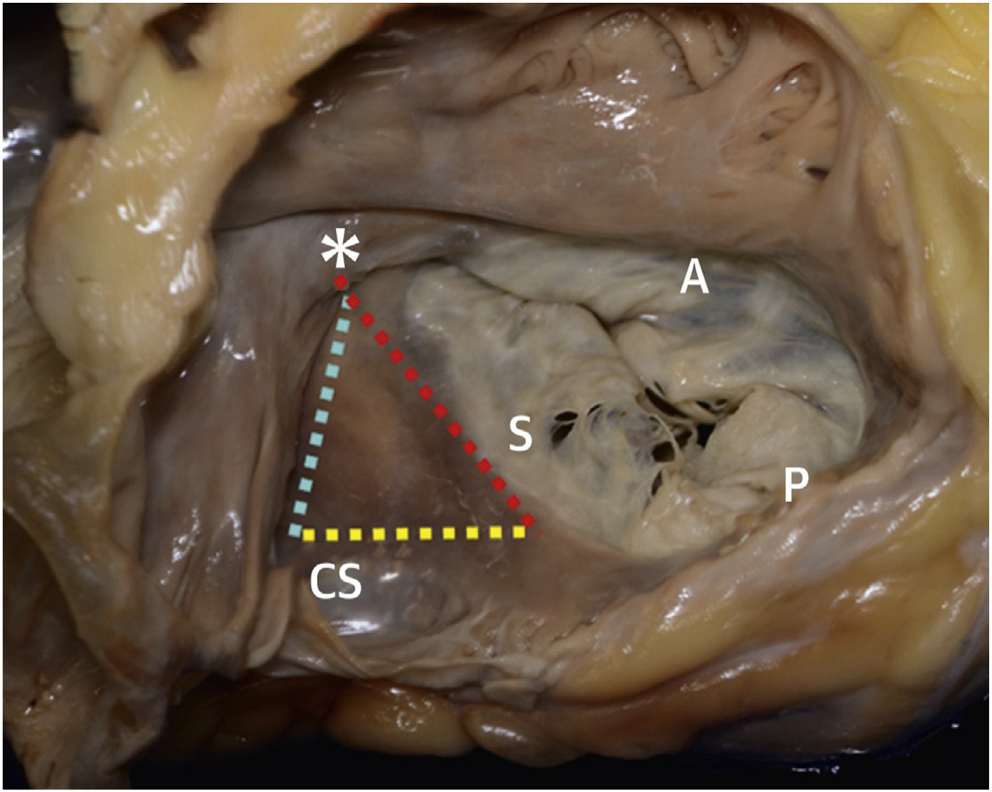
Atrial view demonstrating the tricuspid valve and the proximity of the septal leaflet (*S*) to the AV node (*asterisk*). The *dotted lines* delineate the anatomic landmarks of the triangle of Koch. The tendon of Todaro (*blue line*) lies above the eustachian valve, forming 1 side of the triangle. The hinge point of the septal leaflet (*red line*) forms a second side, and the CS forms the base of the triangle (*yellow line*) with the AV node at the apex of the triangle (*asterisk*). AV = atrio-ventricular; CS = coronary sinus; S = septal leafet; A = anterior leaflet; P = posterior leaflet. Adapted from Elsevier; source: Dahou et al, 2019.^13^

The increased risk of conduction system disease associated with TV intervention is likely attributable to the proximity of the atrioventricular (AV) node to the septal leaflet of tricuspid valve (Figure 1) and its vulnerability to injury during TV intervention. One possible reason for TVr to have consistently lower risk of PPM implantation compared with TVR is that in TV annuloplasty, stitching the annuloplasty ring to the septal portion of the valve or annulus can be avoided to prevent damaging the AV node, whereas in the case of TVR, this area is not spared.

## Transcatheter tricuspid valve intervention (TTVI)

**Figure 2:**
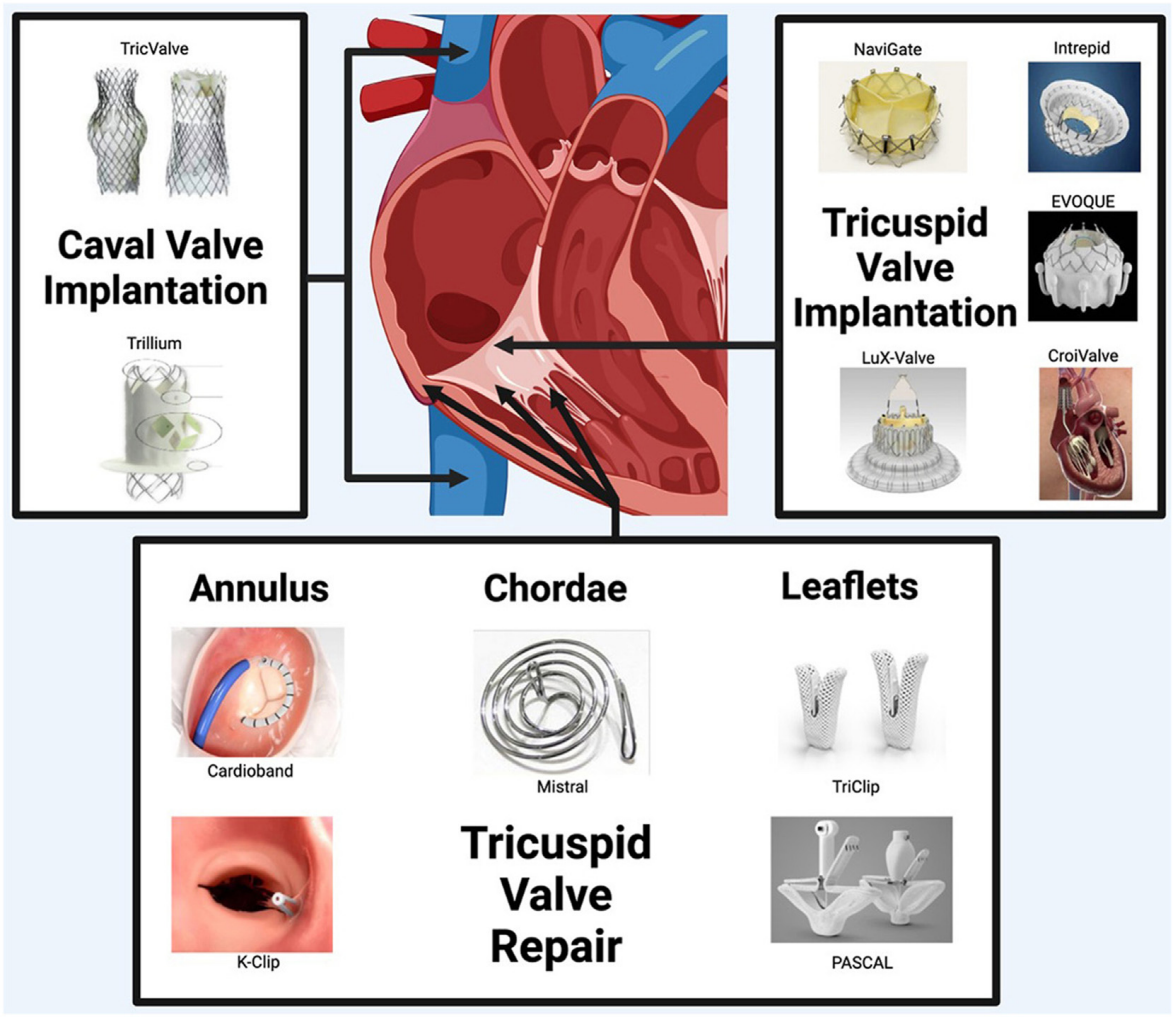
Different transcatheter interventions including replacement and repair. Adapted from Elsevier; source: Seligman et al, 2023.^21^

Despite the increasing volume of procedures performed, mortality rates after TVS remain high at approximately 8%.^6^^,^^14^ In response to this, the use of TTVI for patients with severe symptomatic TR has been rising. Although transcatheter TVR has been associated with significantly lower mortality rates, it is still exposed to high rates of postoperative PPM implantation, ranging from 8% to 11%.^15^, ^16^, ^17^, ^18^ In comparison, the TriValve registry, which investigated transcatheter TVr in 249 patients, found no risk of PPM implantation,^19^ whereas the TRILUMINATE registry, which studied the effects of transcatheter tricuspid edge-to-edge repair (TEER), reported PPM implantation in 2.9% of patients within a year of TEER, although this rate was the same as that for the control group who did not undergo TEER.^20^ Table 1 summarizes the differences in the risk of PPM implantation between interventions. Figure 2 illustrates the different types of TTVI.

**Table 1:** Comparison of PPM implantation rates between interventions

	Surgical TVR	Surgical TVr	Transcatheter TVR	Transcatheter edge-to-edge repair
PPM risk	19%–35%	13%–14%	8%–13%	0%–3%

## Pacing strategies after transcatheter or surgical tricuspid valve replacement or repair

The high rates of PPM implantation after tricuspid valve surgery or intervention highlight the importance of a safe and durable approach to permanent pacing in this cohort. Current European and American guidelines recommend the use of coronary sinus pacing or epicardial pacing after TVR or TVr to avoid crossing the tricuspid valve (Class IIa, grade C recommendation).^22^^,^^23^

### Transvalvular pacing

Studies investigating the use of conventional transvalvular lead implantation after TV intervention have demonstrated mixed outcomes. Current guidelines recommend against transvalvular lead implantation after TVR or TVr.^22^^,^^23^ A retrospective study of 791 patients who underwent surgical TVr found that transvalvular pacemakers implanted preoperatively or within 30 days of surgery were associated with an increased risk of both moderate and severe TR recurrence compared with matched controls without PPM (hazard ratio [HR], 1.6; *P* = .008 and HR, 1.47; *P* = .046, respectively).^24^ Additionally, there was no difference in TR recurrence between the those who had PPM implanted preoperatively and those within 30 days of surgery. The study also found the presence of a transvalvular lead to be a significant predictor of late mortality (HR, 2.12; *P* = .02). Another retrospective study (n = 790) found the presence of a PPM to be a risk factor for late recurrence of at least moderate TR in those undergoing TVr.^25^ Although both studies included patients with preexisting PPM before TVr, the risk of TR recurrence remains higher in those with PPM compared with those without, regardless of the timing of pacemaker implantation. In contrast, Eleid and colleagues^26^ found no difference in TR recurrence after surgical TVR in patients who had postoperative transvalvular leads compared with those without pacemakers (9% vs 5%, respectively; *P* = .45).^26^ However, this was a retrospective study with a smaller sample size (n = 58) and low event rates, and there was a nonsignificant trend towards higher TR recurrence rates in those with transvalvular lead implantation compared with those without. The study also focused on TV replacement rather than repair, and possibly the anatomic durability and robustness of TVR makes the valve less susceptible to valve incompetence in the presence of a transvalvular lead compared with TVr. Overall, limited evidence suggests that the implantation of a transvalvular lead after TVR or TVr should be avoided where possible.

### Coronary sinus pacing

Coronary sinus (CS) pacing involves the implantation of a ventricular lead into 1 or more branches of the coronary sinus to achieve ventricular pacing from the left ventricle.

Small retrospective studies and case reports have demonstrated the safety, feasibility, and durability of single-site coronary sinus pacing (SS-CS) after TV replacement or repair.^27^, ^28^, ^29^, ^30^, ^31^, ^32^ Indeed, in a retrospective analysis of 23 cases of SS-CS matched with transvalvular RV pacing controls, no difference was found in lead parameters or incidence of lead revision or abandonment over a mean follow-up period of 5.3 ± 2.8 years.^31^ Similarly, Li and colleagues^33^ retrospectively analyzed 26 patients who had undergone SS-CS in the context of previous TVS and matched them with transvalvular pacemaker controls.^33^ They found no difference in the rates of lead dysfunction/revision or mortality between the 2 groups. However, compared with the RV pacing group, the CS pacing group had higher pacing impedance (554.69 ohms ± 103.40 vs 784.79 ohms ± 212.72, respectively; *P* < .001) and paced output (0.70V ± 0.17 vs 1.23V ± 0.43, respectively; *P* < .001) at 6 months’ follow-up.

Dual-site CS pacing (DS-CS) is another option that has been adopted after TVR or TVr.^34^, ^35^, ^36^ By having 2 CS leads, DS-CS affords a failsafe in the event of CS lead displacement, which can be important in those who are pacing-dependent. Lee et al^34^ conducted a retrospective analysis of 34 (19 SS-CS and 15 DS-CS) patients who underwent CS-only pacing because of previous TVS.^34^ Two of 19 (10%) SS-CS experienced lead displacement with resultant syncopal episodes, further supporting the use of DS-CS. Notably, a larger retrospective study (n = 847) by Leyva et al^37^ comparing quadripolar and non-quadripolar CS leads found the rate of CS lead displacement to be significantly lower, at 2% for quadripolar leads and 5% for non-quadripolar leads. As well as providing a failsafe, DS-CS has also been demonstrated in a case report to achieve successful cardiac resynchronization therapy with normalization of severe LV systolic dysfunction.^35^ However, larger studies are required to validate the efficacy of DS-CS to achieve CRT. Finally, both epicardial and biventricular pacing have been associated with QT prolongation.^36^ Therefore, QT monitoring during CS pacing is recommended to avoid implanting at a site associated with significant QT prolongation.

### His bundle pacing

His bundle pacing (HBP) achieves biventricular pacing by directly stimulating the bundle of His, which is achievable with lead fixation above or below the tricuspid valve. Special care should be taken during implantation to ensure a supravalvular location of HBP. The atrioventricular septum contains the proximal portion of the His bundle and is situated above the tricuspid annulus. Unipolar mapping here will reveal near-field atrial, His, and ventricular signals. Pacing here will result in paced ventricular activation that is identical to the native His-Purkinje system conduction, and nonselective paraHisian pacing is often not achievable with low outputs because of the paucity of ventricular myocardium in this region.^38^ Implanting in this region is desirable to avoid TV interaction. In contrast, the distal portion of the His bundle, located below the tricuspid annulus, will reveal far-field atrial signals on unipolar mapping with selective His capture only achievable at higher outputs and nonselective capture at low outputs. His bundle pacing here may result in prosthetic valve interaction and should be avoided. Additional measures, such as the use of contrast and 3D transesophageal echocardiogram, have been reported to facilitate the accurate localization of lead tip during HBP.^39^^,^^40^ A small number of case reports describe successful permanent HBP in patients with TVR.^41^, ^42^, ^43^ Rojas and colleagues^42^ describe 2 cases of successful HBP after TVR, 1 in the presence of mechanical TVR and the other in the presence of a bioprosthetic TVR.^44^ In both cases, the HBP threshold was high (4.7 V at 0.5 ms and 3 V at 0.5 ms, respectively), and a backup CS lead was implanted. Although effective at achieving CRT and obviating TV interaction, supravalvular HBP is often associated with high pacing thresholds, poor ventricular sensing, and poor lead stability, making it less suitable as a first-line choice in this scenario.

### Epicardial pacing

Permanent epicardial pacing is often performed during congenital heart disease operations, and temporary epicardial pacing is recommended during TVS (HRS class I, level of evidence C-LD).^23^^,^^44^ However, permanent epicardial pacing is limited by high pacing thresholds and limited lead durability, and patients undergoing transcatheter TV intervention have often been declined for surgical intervention, limiting their suitability for the surgical placement of epicardial leads.^44^^,^^45^ Moreover, in patients with undergoing redo valve operations, the technical feasibility of epicardial pacing is often hampered by adhesions around the ventricles. Although it is reasonable to perform concomitant epicardial pacing in the event of intraoperative significant conduction disease requiring pacing, patients will either require close follow-up to monitor for lead failure or a more reliable backup form of pacing such as CS pacing postoperatively.

### Leadless pacing

The use of leadless pacing (LP) has increased over the last decade. Although its use is currently limited to patients with no upper extremity venous access or at high infection risk based on current guidelines, LP may be a reasonable alternative in those with bioprosthetic TVR or TVr.^5^ The use of LP in patients with prior tricuspid valve surgery is further supported by the recently published Joint American College of Cardiology/American Heart Association/American Society of Echocardiography/Heart Rhythm Society/Society of Cardiovascular Angiography and Interventions/Society of Cardiovascular Computed Tomography/Society for Cardiovascular Magnetic Resonance 2025 (ACC/AHA/ASE/HFSA/HRS/SCAI/SCCT/SCMR2025) Appropriate Use Criteria for Implantable Cardioverter-Defibrillators, Cardiac Resynchronization Therapy and Pacing, and the 2022 position paper on leadless cardiac implantable electronic devices (CIED) by European Heart Rhythm Association/Heart Rhythm Society/Latin American Heart Rhythm Society/Asia Pacific Heart Rhythm Society (EHRA/HRS/LAHRS/APHRS).^46^^,^^47^ By removing the presence of a lead, LP circumvents any ongoing interaction with the TV and obviates the risks associated with pocket infection and lead fracture.

LP has been shown to reduce the risk of TR compared with conventional transvalvular pacing. In a large multicenter registry of Micra LP (Medtronic, Minneapolis, MN) (n = 1809), the rate of TR at 5-year follow-up was 0.06%.^48^ However, the current delivery sheaths remain relatively large-bore (23F for Micra and 25F for AVEIR [Abbott, Chicago, IL]), and it is unclear how this would affect TV prostheses during implantation. Small retrospective studies have described acute success, safety, and durability of LP in the context of bioprosthetic TVR or TVr.^49^, ^50^, ^51^, ^52^ Theis et al^51^ conducted a retrospective analysis of 14 consecutive patients who underwent early postoperative LP (Micra) implantation after bioprosthetic TVR or TVr. Echocardiography immediately after LP implantation showed no change in TV function before implantation, and LP pacing parameters remained stable at 6-month follow-up. In addition, 2 patients who had early LP implantation after concomitant mitral valve surgery for infective endocarditis showed no signs of reinfection at follow-up, a finding that is supported by other studies, including that by Beurskens et al^52^ who found no reinfection in patients who had undergone LP implantation after infected transvenous system extractions, with a mean follow-up of 16 ±12 months.^52^^,^^53^ Similarly, a multicenter analysis of LP (Micra) implantation in patients who had undergone bioprosthetic TVR or TVr demonstrated no difference in the primary composite outcome of severe TR development, death, or CRT upgrade.^49^

Overall, long-term data on the use of LP after TV surgery or intervention are lacking. Evidence from small retrospective studies suggests LP implantation in this cohort is safe, does not affect TV function, and is not associated with device reinfections when implanted early after infective endocarditis treatment. LP should be considered in patients with bioprosthetic TVR or TVr who are at high risk of infection, such as those with recent history of infective endocarditis, or who are on long-term hemodialysis, if a PPM indication arises after surgery.

**Figure 3:**
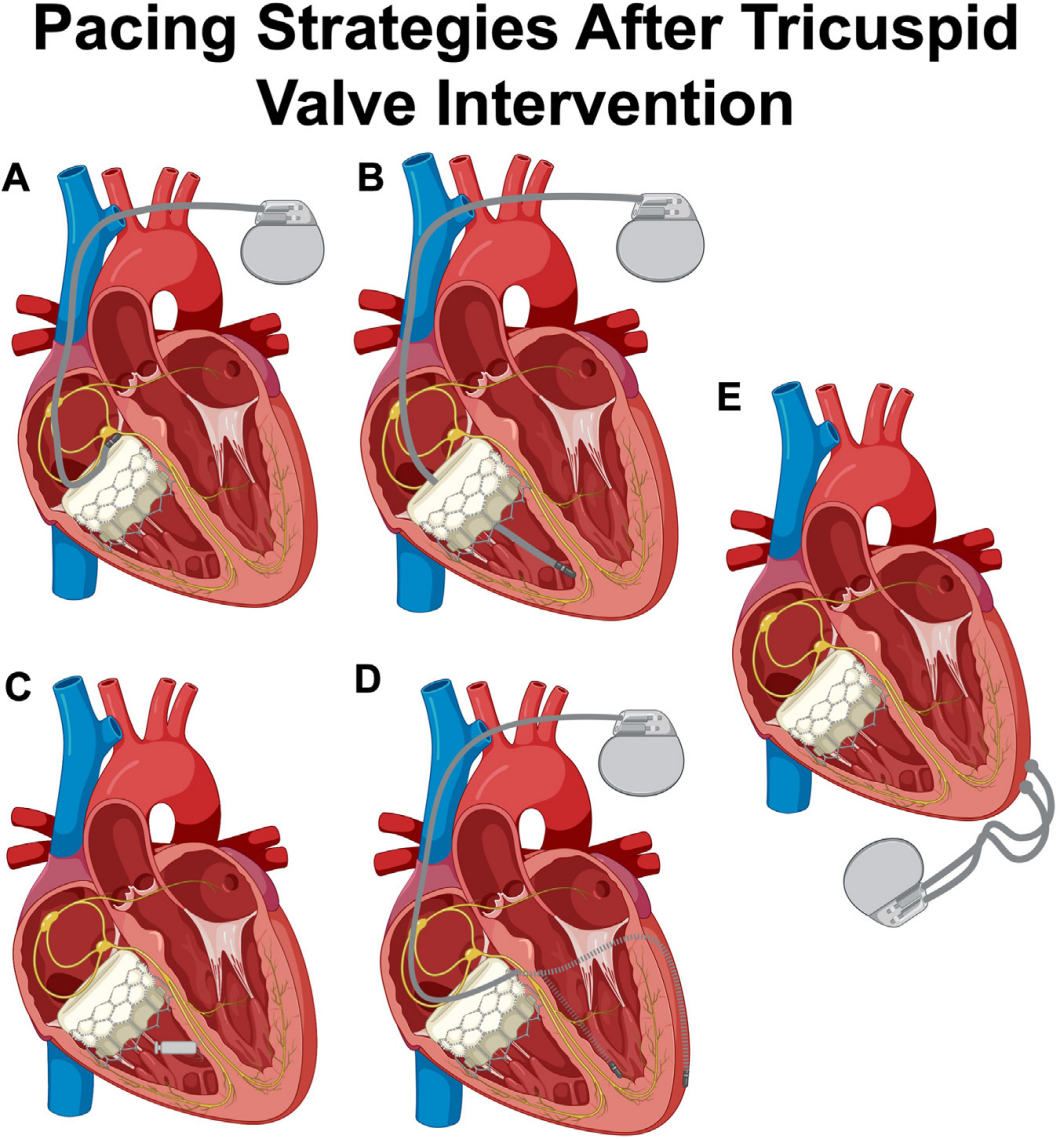
Different ventricular pacing strategies after TVR. **A:** His bundle pacing; **B:** Transvalvular RV pacing; **C:** Leadless pacing; **D:** DS-CS, dashed portion of lead to indicate location posterior to the heart; **E:** Epicardial pacing. RV = right ventricular; DS-CS = dual-site coronary sinus pacing.

Figure 3 illustrates the different pacing strategies after TVR. Table 2 compares the advantages and disadvantages of different pacing strategies after TV intervention.

**Table 2:** Advantages and disadvantages of different pacing strategies after TVR or TVr

Advantages	Pacing strategy	Disadvantages
Extensive data on efficacy and durability in general population	Transvalvular pacing	Associated with increased moderate or severe TR recurrence and increased late mortality
No TV interaction. Good durability and lead failure/revision rates comparable to conventional transvalvular leads DS-CS option affords failsafe in the event of lead dislodgement and may also offer resynchronization therapy	CS pacing	Limited by suitability of CS anatomy Higher risk of QT prolongation due to epicardial position
No TV interaction. Physiologic ventricular activation may reduce risk of pacing-induced cardiomyopathy	His bundle pacing	High thresholds and poor lead stability
No TV interaction Can be performed concomitantly during TVS	Epicardial pacing	Poor lead durability May be technically challenging in patients with previous open-heart surgery caused by adhesions around ventricle
No ongoing TV interaction Low risk of device infection compared with transvenous strategies	Leadless pacing	Impact of large-caliber delivery sheaths on TV unclear Extraction of leadless device in the event of infection may worsen TV function Ongoing uncertainty regarding management of device at the end of battery life

## Defibrillation therapy after transcatheter or surgical tricuspid valve replacement or repair

The large caliber of transvenous implantable cardiac defibrillator (ICD) leads and the requirement for effective shocking vectors represent a challenge for ICD implantation after TVR or TVr. ICD leads are also contraindicated in those with mechanical TVR.^22^^,^^23^ To circumvent the crossing of TV, other methods of transvenous ICD implantation have been tried, including placing the ICD lead into the middle cardiac vein, lateral branch of the coronary sinus, azygous vein, low right atrium, and inferior vena cava.^28^^,^^54^, ^55^, ^56^, ^57^, ^58^ Extravascular options include subcutaneous, extravascular, and epicardial ICD implantation.

### Subcutaneous ICD

The use of subcutaneous ICD (S-ICD) avoids interaction with the TV. However, S-ICD does not allow for bradycardia pacing, resynchronization therapy, or anti-tachycardia pacing (ATP). This is particularly pertinent because a significant proportion of patients undergoing TV intervention will require PPM, and many will require a high burden of ventricular pacing secondary to high-grade AV block. The use of S-ICD is also contingent on the passing of S-ICD screening. Thus, S-ICD implantation should mainly be considered in patients with TVR or TVr at risk of ventricular arrhythmias who are at low risk of requiring bradycardia pacing. In cases in which there is a pacing indication or frequent sustained ventricular tachycardia, the use of S-ICD should be combined with other forms of pacing, such as LP or CS pacing, or an alternative form of pacemaker-defibrillator should be considered entirely, such as extravascular ICD (EV-ICD). The MODULAR ATP study investigating the combination of the EMPOWER (Boston Scientific) LP and EMBLEM (Boston Scientific). S-ICD has shown significant promise and may be an attractive alternative, when it is commercially available, where there is a concurrent need for bradycardia pacing and ATP.^59^

### EV-ICD

An EV-ICD involves the substernal implantation of the shocking lead and has a smaller pulse generator than the S-ICD system. Because of the substernal position of the shocking lead, the EV-ICD is able to deliver ATP and pause-prevention pacing and is expected to have a longer battery life because of the lower energy required for defibrillation. However, the use of EV-ICD may be limited in those with prior sternotomies because of the requirement for substernal implantation of the shocking lead. The global EV-ICD Pivotal study demonstrated the safety and efficacy of the EV-ICD over a 6-month period.^60^ Notably, the first-shock efficacy of the EV-ICD was lower (78%), and the rate of inappropriate shock was higher (8.5%) compared with current transvenous and subcutaneous ICDs. The overall defibrillation efficacy was equal to or greater than transvenous and subcutaneous ICDs. Five percent of patients (14 of 284) had ATP turned off or not programmed because of the pacing sensation not being acceptable.

### Prevalvular transvenous ICD

**Figure 4:**
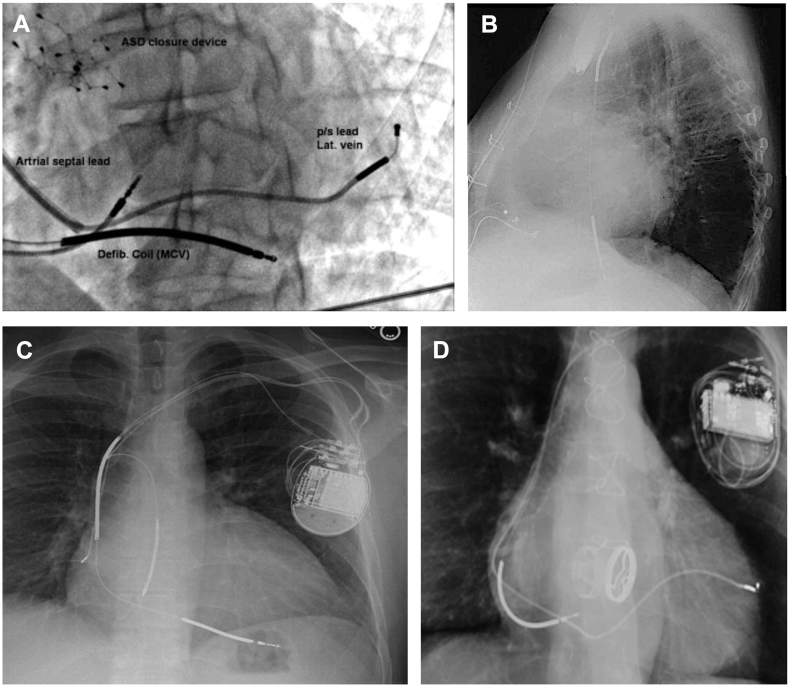
Different approaches to prevalvular transvenous ICD implantation. **A:** ICD in MCV and pace/sense lead in posterolateral vein of CS (Adapted from Elsevier; Source: Lopez et al, 2009^55^); **B:** Floating ICD in IVC visible on lateral chest radiograph (Adapted from John Wiley and Sons; Source: Schreiber et al, 2003^56^); **C:** Additional ICD in azygous vein (pictured along the spine) on postero-anterior chest radiograph (Adapted from Elsevier; Source: Cooper et al, 2009^61^); **D:** ICD in low right atrium with coronary sinus lead in posterior branch of CS (Adapted from John Wiley and Sons; Source: Biffi et al, 2006^57^).

The implantation of ICD in the coronary sinus has been reported. Srinivasan and Segal^54^ described a case of ICD lead implantation in the mid lateral branch of the CS in a patient with mechanical TVR, reduced left ventricular ejection fraction of 40% and symptomatic monomorphic ventricular tachycardia.^54^ Because of a failure of S-ICD screening and the need for resynchronization therapy, 2 leads, including 1 ICD lead, were implanted in separate branches of the coronary sinus. This allowed for biventricular pacing and ATP to be delivered with successful reduction in QRS duration. Lopez et al^55^^,^^58^ reported similar cases of biventricular ICD pacing, with the ICD lead positioned in the middle cardiac vein (Figure 4A).^55^^,^^58^ In 1 case, the defibrillation threshold was higher than normal.

Although feasible, the standard CS delivery sheaths cannot accommodate the larger ICD leads and can make CS cannulation technically challenging. Because of the larger caliber of ICD leads, the risk of CS perforation is increased, and the number of CS branches that can accommodate the ICD lead may be limited. Therefore, operators undertaking ICD implantation in the CS should be experienced and comfortable with the use of nonstandard delivery sheaths to engage the CS. A further factor to be taken into consideration is the risk associated with ICD lead extraction from the CS. Although small studies have demonstrated the safety of CS pace/sense lead extraction, CS ICD lead extraction has rarely been performed. Hamid et al^62^ described the removal of a defibrillator lead buried in the CS requiring the use of laser within the CS.^62^

Separately, 2 case reports have described ICD lead implantation in the inferior vena cava (IVC) after mechanical and bioprosthetic TVR, respectively.^28^^,^^56^ In both cases, the ICD lead was left floating in the IVC (Figure 4B). Defibrillation thresholds were satisfactory in both cases, although 1 report described a rising but acceptable pacing threshold at follow-up (<0.6 V–1.4 V at 0.5 ms).

Biffi et al^57^ described a case of implanting an ICD lead with active fixation in the low right atrium after mechanical TVR (Figure 4D).^57^ This yielded satisfactory defibrillation threshold, and lead parameters remained stable after 16 months. Figure 4 illustrates the different approaches to prevalvular transvenous ICD implantation.

Overall, evidence around the safety, efficacy, and durability of prevalvular transvenous ICD is sparse, and this approach should only be considered if other, more established methods are not available.

### Epicardial ICD

Although data on the use of epicardial ICD after TVR or TVr are extremely limited, several case reports and case series have described epicardial ICD implantation in other etiologies.^63^ A case series of 11 adult patients with heterogenous etiology who had undergone epicardial ICD/CRT-D at our institution found 54% (6 of 11) requiring surgical or percutaneous reintervention for device-related complications.^63^ A systematic review of case reports and case series of epicardial ICD/CRT-D (n = 188) found the most common indication for epicardial device was young age (48%), followed by lack of venous access(14%). Of these, 93% had successful defibrillation threshold testing. Overall, the risk of lead-related complication requiring reintervention is high with epicardial ICD implantation, but it may be a reasonable option for primary prevention in patients already undergoing concomitant open-heart surgery.

## Cardiac resynchronization therapy after transcatheter or surgical tricuspid valve replacement or repair

Current guidelines recommend that CRT should be considered in patients with anticipated high ventricular pacing percentage and left ventricular ejection fraction <50%.^22^^,^^23^ As described earlier, biventricular pacing in the form of DS-CS has been attempted with successful QRS shortening. However, whether long-term outcomes of DS-CS are comparable to conventional transvenous CRT will need to be validated in larger studies.

Resynchronization therapy in the form of LP has also been performed using the WISE-CRT device (EBR Systems, Sunnyvale, CA).^59^ This is a 2-part system consisting of an ultrasound transmitter containing a battery that is inserted subcutaneously between the 4^th^ and 6^th^ intercostal space and a receiver electrode that is implanted in the left ventricle via transseptal or transaortic access. The WISE-CRT system relies on the detection of a pacing stimulus by ‘co-implant’ such as a transvenous RV lead or leadless RV pacemaker. After detection of stimulus from the co-implant, the ultrasound transmitter produces a series of short ultrasound pulses to align with the receiver followed by longer ultrasound pulses that are converted to pacing stimulus in the left ventricle, thus achieving CRT. Carabelli et al^64^ investigated the implantation of the WISE-CRT system in 8 patients who already had a right ventricular LP (Micra) who developed pacing-induced cardiomyopathy and were not suitable for conventional CRT vecause of infection risk or anatomic condition.^64^ In this study, WISE-CRT devices were able to detect Micra pacing output in all cases and deliver synchronous LV pacing with significant reduction in QRS duration (pre-CRT mean QRS, 204.38 ± 30.26 vs post-CRT, 137.5 ± 24.75 ms; *P* = .012). The recently published SOLVE-CRT study further reinforced the efficacy of the WISE-CRT system in achieving positive remodeling in conventional CRT nonresponders, those who had failed attempt at conventional CRT, and those deemed too high risk for CS lead implant.^65^

## Lead extraction after transcatheter or surgical tricuspid valve intervention

### Jailed or entrapped transvenous leads

Up to one third of patients have preexisting transvenous right ventricular pacing leads before undergoing surgical or transcatheter TVR or TVr.^66^^,^^67^ Although the American guidelines do not currently offer any recommendations on this subject, the European guidelines recommend the removal of transvalvular leads before TVR followed by reimplantation of an alternative pacing system.^22^^,^^23^ This pre-empts the high-risk and technically challenging removal of jailed leads in the event of device infection.

**Figure 5:**
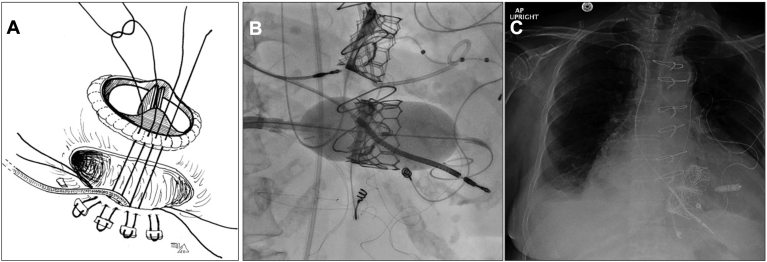
**A:** Mattress stitches are placed on either side of the lead during surgical TVR, thus entrapping the lead between TV prosthesis and TV annulus. (Adapted from Elsevier; Source: Molina et al, 2010^68^). **B:** The ballooning of the TVIV prosthesis over the transvalvular lead, thus jailing it. **C:** Remnant of jailed RV ICD lead after incomplete lead extraction. The lead was cut at the venous access site, and an LP was subsequently implanted in the RV. **B** and **C** were adapted from John Wiley and Sons; Source: Rios et al, 2020.^76^

Despite this, the entrapment or jailing of leads outside of the new valve during TVS or TTVIV is performed.^15^^,^^68^, ^69^, ^70^, ^71^, ^72^, ^73^, ^74^ Figure 5A and 5B depicts the entrapment of leads during surgical TVR and TTVIV, respectively. Case reports of this practice in surgical TVR suggest no long-term detrimental effect on lead function.^68^, ^69^, ^70^ In contrast, a retrospective study of jailed leads during TTVIV (n = 28) found a 11% rate of lead complication requiring intervention (4% lead failure, 7% lead dislodgement) during a median follow-up of 15.2 months, which is significantly higher than the rate of lead failure in the general adult population.^75^ The valve-in-valve international database registry reported a similar rate of 11% lead complication requiring intervention after lead jailing during TTVIV.^67^

Limited case reports describe the removal of jailed leads. Bhuta et al^77^ described the removal of a transvalvular lead jailed by a bioprosthetic valve. An initial attempt to free the jailed lead by advancing a laser sheath close to the TV annulus was unsuccessful, and subsequently, a femoral snaring technique was used to remove the lead, with no paravalvular leak observed. Kypta et al^74^ described extraction of a lead jailed by a mechanical TVR by advancing a lead-locking device up to the mechanical valve ring, followed by countertraction, resulting in successful detachment of the lead from the ventricular wall.^74^ In contrast, Rios et al^76^ described a case of incomplete extraction of a lead jailed by TTVIV in the context of infective endocarditis.^76^ Because of the perceived risk of TTVIV dislodgment and embolization, the RV lead was cut and capped at the venous access site, leaving a remnant RV lead from the subclavian down to the RV apex (Figure 5C).

Extraction of jailed leads is technically challenging and high-risk because of several factors. Removal of jailed leads may result in displacement and embolization of transcatheter TVR or, in the case of surgical TVR, significant paravalvular leak may occur because of the gap left behind by the removed lead. The wide caliber of extraction tools may increase the risk of damaging the valve replacement or TV annulus, and in cases in which lead dwell time is prolonged and the lead is embedded in the myocardium at the level of the TV annulus, traction and countertraction applied during extraction may not be transmitted beyond the annulus and may result in snapping of leads, leaving lead remnants in the right ventricle, which would require high-risk surgical removal. Finally, during extraction of leads with vegetation distal to the TV annulus, there is a risk of vegetation embolization. For these reasons, extraction of leads before surgical or transcatheter TVR should be preferred, with lead jailing to be considered only where lead extraction is deemed too high risk, ICD therapy is not required, or the anticipated pacing burden is low.^78^

## Practical recommendations

**Figure 6:**
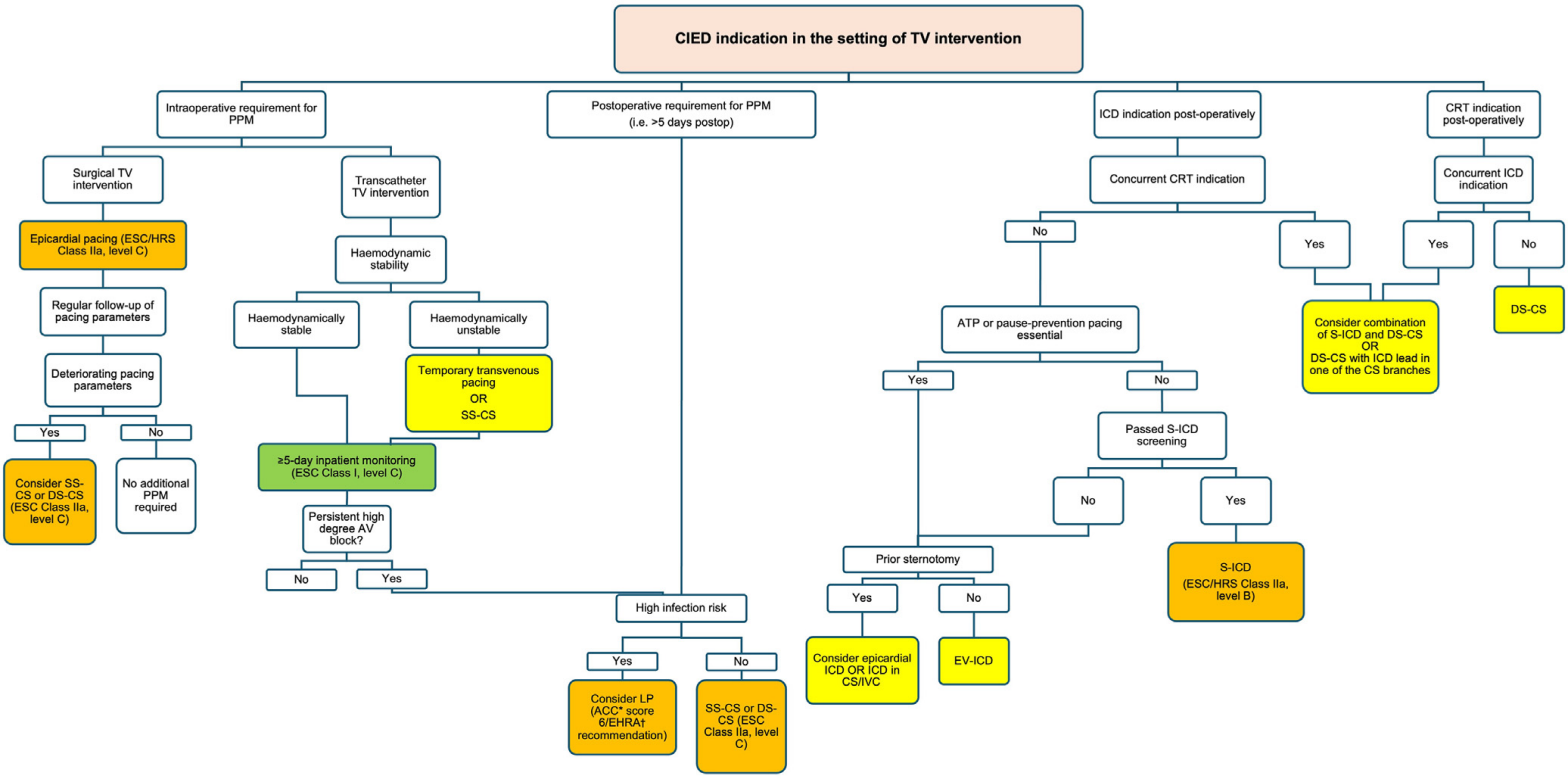
Proposed algorithm for CIED indication during or after TV intervention. Where available, the level of evidence and degree of recommendation is listed alongside the intervention. *Boxes* shaded green denote a class I indication, amber denotes a class IIa indication, and yellow denotes the absence of any formal recommendation. CIED = Cardiac implantable electronic devices; ESC = European Society of Cardiology; HRS = Heart Rhythm Society; SS-CS = single-site coronary sinus pacing; DS-CS = dual-site coronary sinus pacing; EV-ICD = extravascular implantable cardiac defibrillator; S-ICD = subcutaneous implantable cardiac defibrillator; CS = coronary sinus. ∗ACC = ACC/AHA/ASE/HFSA/HRS/SCAI/SCCT/SCMR 2025 Appropriate Use Criteria for Implantable Cardioverter-Defibrillators. Score 6 denotes ‘May Be Appropriate’ indication. EHRA† = EHRA/HRS/LAHRS/APHRS position paper on leadless cardiac implantable electronic devices.

Based on currently available data on CIED implantation in the context of TV replacement or repair, we propose an algorithm for CIED implantation during or after TV intervention, summarized in Figure 6.

### Bradycardia pacing during or after TV intervention

Where an intraoperative requirement for pacing arises during surgical TV intervention, we recommend epicardial pacing, as per international guidelines.^22^^,^^23^ However, because of the poor durability of epicardial pacing, we recommend close follow-up of pacing parameters to inform the need for a more durable pacing strategy that avoids TV interaction, such as SS-CS or DS-CS. For patients with a pacing requirement during transcatheter TV intervention and who are hemodynamically unstable, we recommend either temporary transvalvular pacing, except in the case of mechanical TVR where this is contraindicated, or temporary SS-CS using a standard CS lead that is then connected to a temporary pacing box. In those who are hemodynamically stable, we recommend a minimum of 5 days of observation to assess for resolution of transient high-grade AV block, consistent with current guidelines.^22^^,^^23^ If high-grade AV block is persistent after a period of monitoring, SS-CS or DS-CS should be considered if device infection risk is low. If the infection risk is high, we believe the use of LP is a reasonable alternative, given the respective recommendations on its use in those at high risk of infection and those who have undergone previous TV surgery, by the ESC (Class IIa, level B) and ACC/AHA/ASE/HFSA/HRS/SCAI/SCCT/SCMR (score 6), respectively.^22^^,^^46^

### Defibrillator or cardiac resynchronization therapy after TV intervention

Where an indication for defibrillator therapy arises after TV intervention with no concurrent CRT indication, S-ICD implantation, provided screening is successful, is an option. Where ATP or pause-prevention pacing is essential, or S-ICD screening is unsuccessful, EV-ICD may be considered in the absence of prior sternotomy. Other options include epicardial ICD or implantation of ICD in the CS or IVC, with the caveat that safety, efficacy, and durability data on these strategies are very limited. Where there is a concurrent CRT indication, a combination of S-ICD and DS-CS or DS-CS alone with an ICD lead and a pace/sense lead in different branches of the coronary sinus may be considered, with the caveat that the latter option is largely evidence-free. Where there is an indication for CRT after TV intervention, with no concurrent need for defibrillator therapy, we would recommend DS-CS as an attempt at achieving biventricular pacing without crossing the TV, recognizing the paucity of evidence in this area.

## Future perspectives

With the emerging favorable safety profile of transcatheter tricuspid interventions, likely more transcatheter procedures will be performed for the treatment of isolated TR in the future. Current transcatheter TVR methods are associated with a significant risk of PPM implantation postprocedure. With the promising results of transcatheter TVr, there may be a shift toward repair over replacement, which is associated with significantly lower risk of PPM implantation.

Currently, limited data are available regarding the safety of LP in the context of TVR and TVr, although case reports have demonstrated favorable outcomes.^49^, ^50^, ^51^ With the rapidly increasing prevalence of LP implantation, we anticipate the safety profile of LP in this context to be established in the next few years.

Although S-ICD is a good option for defibrillator therapy without affecting TV function after TVR or TVr, it does not allow for bradycardia pacing, resynchronization therapy, or ATP. The investigational Boston Scientific EMPOWER LP seeks to address this by offering a modular cardiac rhythm management system in which the EMPOWER LP can communicate with the EMBLEM S-ICD to provide bradycardia pacing, ATP, and defibrillator therapy. Being a modular system, the EMPOWER LP does not have to be implanted simultaneously with an ICD and is compatible with previously implanted EMBLEM S-ICDs.

Although there is currently no licensed option for a completely leadless CRT/D system, the concurrent use of the Micra LP together with the investigational WISE-CRT system to provide completely leadless CRT has been described by Carabelli and colleagues^64^ with significant QRS reduction. In addition, Sidhu and colleagues^79^ reported the successful implantation of completely leadless CRT-D system with the Micra LP, EMBLEM S-ICD, and WISE-CRT system.^79^ This, however, involved the use of 3 different systems, each by a different manufacturer, and because of the lack of integration between the Micra LP and EMBLEM S-ICD, ATP is not possible. The combination of the integrated Boston Scientific S-ICD and LP system, together with the WISE-CRT system, may be possible in the near future, subject to regulatory approval for the latter, and may provide a less fragmented approach to delivering leadless ATP, cardiac resynchronization, and defibrillator therapy.

The jailing of transvalvular leads during surgical or transcatheter TVR is likely to increase with the increasing number of TVR performed. Given the technical difficulty and complexity of lead extraction in these instances, better education regarding the impact of jailed leads on future extractions and joint decision-making between electrophysiologists, structural interventionalists, and cardiac surgeons in the form of multidisciplinary meetings before TVR and TVr, factoring in the individual’s wishes, should be strongly encouraged.

## Conclusion

The risk of conduction system disease requiring PPM implantation remains high after TV intervention. Reasonable options of permanent pacing in this cohort that avoids TV interaction include CS pacing, epicardial pacing, and LP, depending on the circumstances. Where defibrillator therapy is required, the use of S-ICD or EV-ICD is preferred after TVR or TVr to avoid TV interaction, depending on the presence of previous sternotomy, because transvenous prevalvular ICD and epicardial ICD lack safety and durability data. The jailing of transvalvular leads during TVR should be avoided unless the risk of extraction is high. A multidisciplinary approach should be adopted routinely to discuss pacing strategies and lead extraction in the context of TVR or TVr. Future perspectives regarding a completely leadless CRT-D system are promising and may be the way forward for patients with previous TV intervention.

## References

[1] Wang N., Fulcher J., Abeysuriya N. (2019). Tricuspid regurgitation is associated with increased mortality independent of pulmonary pressures and right heart failure: a systematic review and meta-analysis. Eur Heart J [Internet].

[2] Taramasso M., Benfari G., van der Bijl P. (2019). Transcatheter versus medical treatment of patients with symptomatic severe tricuspid regurgitation. J Am Coll Cardiol.

[3] Offen S., Playford D., Strange G., Stewart S., Celermajer D.S. (2022). Adverse prognostic impact of even mild or moderate tricuspid regurgitation: insights from the national echocardiography database of Australia. J Am Soc Echocardiogr.

[4] Otto C.M., Nishimura R.A., Bonow R.O. (2021). 2020 ACC/AHA Guideline for the management of patients with valvular heart disease: a report of the American College of Cardiology/American Heart Association Joint Committee on Clinical Practice Guidelines. Circulation [Internet] American Heart Association.

[5] Vahanian A., Beyersdorf F., Praz F. (2022). 2021 ESC/EACTS Guidelines for the management of valvular heart disease: developed by the Task Force for the management of valvular heart disease of the European Society of Cardiology (ESC) and the European Association for Cardio-Thoracic Surgery (EACTS). Eur Heart J [Internet].

[6] Zack C.J., Fender E.A., Chandrashekar P. (2017). National trends and outcomes in isolated tricuspid valve surgery. J Am Coll Cardiol.

[7] Alqahtani F., Berzingi C.O., Aljohani S., Hijazi M., Al-Hallak A., Alkhouli M. (2017). Contemporary trends in the use and outcomes of surgical treatment of tricuspid regurgitation. J Am Heart Assoc American Heart Association.

[8] Kassab J., Harb S.C., Desai M.Y. (2024).

[9] Gammie J.S., Chu M.W.A., Falk V. (2022). Concomitant tricuspid repair in patients with degenerative mitral regurgitation. N Engl J Med.

[10] Jokinen J.J., Turpeinen A.K., Pitkänen O., Hippeläinen M.J., Hartikainen J.E.K. (2009). Pacemaker therapy after tricuspid valve operations: implications on mortality, morbidity, and quality of life. Ann Thorac Surg.

[11] Kho J., Ioannou A., O’Sullivan K.E., Jones M. (2020). Permanent pacemaker implantation rates following cardiac surgery in the modern era. Irish J Med Sci.

[12] Mar P.L., Angus C.R., Kabra R. (2017). Perioperative predictors of permanent pacing and long-term dependence following tricuspid valve surgery: a multicentre analysis. Europace.

[13] Dahou A., Levin D., Reisman M., Hahn R.T. (2019). Anatomy and physiology of the tricuspid valve. JACC Cardiovasc Imaging.

[14] Kilic A., Saha-Chaudhuri P., Rankin J.S., Conte J.V. (2013). Trends and outcomes of tricuspid valve surgery in North America: an analysis of more than 50,000 patients from the Society of Thoracic Surgeons database. Ann Thorac Surg.

[15] Fam N.P., von Bardeleben R.S., Hensey M. (2021). Transfemoral transcatheter tricuspid valve replacement with the EVOQUE system: a multicenter, observational, first-in-human experience. JACC Cardiovasc Interv.

[16] Zhang Y., Lu F., Li W. (2023). A first-in-human study of transjugular transcatheter tricuspid valve replacement with the LuX-Valve Plus system. EuroIntervention Europa Group.

[17] Mao Y., Li L., Liu Y. (2022). Safety, efficacy, and clinical outcomes of transcatheter tricuspid valve replacement: one-year follow-up. Front Cardiovasc Med.

[18] Kodali S., Hahn R.T., Makkar R. (2023). Transfemoral tricuspid valve replacement and one-year outcomes: the TRISCEND study. Eur Heart J.

[19] Mehr M., Taramasso M., Besler C. (2019). 1-Year outcomes after edge-to-edge valve repair for symptomatic tricuspid regurgitation: results from the TriValve Registry. JACC Cardiovasc Interv.

[20] Paul S., Brian W., Nadira H. (2023). Transcatheter repair for patients with tricuspid regurgitation. N Engl J Med.

[21] Seligman H., Vora A.N., Haroian N.Q. (2023). The current landscape of transcatheter tricuspid valve intervention. J Soc Cardiovasc Angiogr Interv.

[22] Glikson M., Nielsen J.C., Kronborg M.B. (2021). 2021 ESC guidelines on cardiac pacing and cardiac resynchronization therapy: developed by the Task Force on cardiac pacing and cardiac resynchronization therapy of the European Society of Cardiology (ESC) with the special contribution of the European Heart Rhythm Association (EHRA). Eur Heart J.

[23] Kusumoto F.M., Schoenfeld M.H., Barrett C. (2019). 2018 ACC/AHA/HRS guideline on the evaluation and management of patients with bradycardia and cardiac conduction delay: a report of the American College of Cardiology/American Heart Association Task Force on Clinical Practice Guidelines and the Heart Rhythm Society. Circulation.

[24] Mazine A., Bouchard D., Moss E. (2013). Transvalvular pacemaker leads increase the recurrence of regurgitation after tricuspid valve repair. Ann Thorac Surg.

[25] McCarthy P.M., Bhudia S.K., Rajeswaran J. (2004). Tricuspid valve repair: durability and risk factors for failure. J Thorac Cardiovasc Surg.

[26] Eleid M.F., Blauwet L.A., Cha Y.M. (2012). Bioprosthetic tricuspid valve regurgitation associated with pacemaker or defibrillator lead implantation. J Am Coll Cardiol.

[27] Sideris S., Drakopoulou M., Oikonomopoulos G. (2016). Left ventricular pacing through coronary sinus is feasible and safe for patients with prior tricuspid valve intervention. Pacing Clin Electrophysiol.

[28] Grimard C., Clémenty N., Fauchier L., Babuty D. (2010). Ventricular pacing through coronary sinus in patients with tricuspid prosthesis. Ann Thorac Surg.

[29] Yoda M., Nakai T., Okubo K. (2008). First case report in Japan of left ventricular pacing via a coronary vein in a patient with a mechanical tricuspid valve. Circ J.

[30] Liu T.I., Feld G.K., Birgersdotter-Green U. (2008). Transvenous left ventricular lead placement in a patient with mechanical tricuspid, aortic, and mitral valves. Eur Heart J.

[31] Noheria A., Van Zyl M., Scott L.R. (2018). Single-site ventricular pacing via the coronary sinus in patients with tricuspid valve disease. EP Europace.

[32] Bai Y., Strathmore N., Mond H., Grigg L., Hunt D. (1994). Permanent ventricular pacing via the great cardiac vein. Pacing Clin Electrophysiol.

[33] Li T.Y.W., Seow S.C., Singh D., Yeo W.T., Kojodjojo P., Lim T.W. (2019). Left ventricular pacing in patients with preexisting tricuspid valve disease. J Arrhythm.

[34] Lee C.C., Do K., Patel S. (2019). Single- and dual-site ventricular pacing entirely through the coronary sinus for patients with prior tricuspid valve surgery. J Interv Cardiac Electrophysiol.

[35] Salcedo J., Doshi R.N. (2012). Successful cardiac resynchronization defibrillator placement in a patient with tetralogy of Fallot, widened QRS duration, and mechanical tricuspid valve replacement using dual-site left ventricular venous lead placement. J Innov Card Rhythm Manag.

[36] Medina-Ravell V.A., Lankipalli R.S., Yan G.X. (2003). Effect of epicardial or biventricular pacing to prolong QT interval and increase transmural dispersion of repolarization: does resynchronization therapy pose a risk for patients predisposed to long QT or torsade de pointes?. Circulation.

[37] Leyva F., Zegard A., Qiu T. (2017). Cardiac resynchronization therapy using quadripolar versus non-quadripolar left ventricular leads programmed to biventricular pacing with single-site left ventricular pacing: impact on survival and heart failure hospitalization. J Am Heart Assoc.

[38] Nagarajan V.D., Ho S.Y., Ernst S. (2019). Anatomical considerations for His bundle pacing. Circ Arrhythm Electrophysiol.

[39] von Alvensleben J.C., Runciman D.M., Schaffer M., Burkett D., Jone P.-N., Collins K.K. (2023). Use of adjunctive 3-dimensional echocardiogram for His bundle pacing in pediatric patients. HeartRhythm Case Rep.

[40] Gu M., Niu H., Hu Y. (2020). Permanent His bundle pacing implantation facilitated by visualization of the tricuspid valve annulus. Circ Arrhythm Electrophysiol.

[41] Rojas S.C.F., Schurmann P.A., Rodríguez-Mañero M., Lustgarten D., Valderrábano M. (2019). Permanent His-bundle pacing from the right atrium in patients with prosthetic tricuspid valve. HeartRhythm Case Rep.

[42] Torres-Quintero L., Molina-Lerma M., Cabrera-Borrego E., García-Orta R., Tercedor-Sánchez L., Álvarez M. (2020). His-bundle pacing from the right atrium in a patient with tetralogy of fallot and a prosthetic tricuspid valve. Clin Electrophysiol.

[43] Sharma P.S., Subzposh F.A., Ellenbogen K.A., Vijayaraman P. (2017). Permanent His-bundle pacing in patients with prosthetic cardiac valves. Heart Rhythm.

[44] McLeod C.J., Jost C.H.A., Warnes C.A. (2010). Epicardial versus endocardial permanent pacing in adults with congenital heart disease. J Interv Cardiac Electrophysiol.

[45] Cooper J.P., Jayawickreme S.R., Swanton R.H. (1995). Permanent pacing in patients with tricuspid valve replacements. Br Heart J.

[46] Russo A.M., Desai M.Y., Do M.M. (2025). ACC/AHA/ASE/HFSA/HRS/SCAI/SCCT/SCMR 2025 Appropriate use criteria for implantable cardioverter-defibrillators, cardiac resynchronization therapy, and pacing [Published online ahead of print January 13, 2025]. J Am Coll Cardiol.

[47] Boersma L.V., El-Chami M., Steinwender C. (2022). Practical considerations, indications, and future perspectives for leadless and extravascular cardiac implantable electronic devices: a position paper by EHRA/HRS/LAHRS/APHRS. EP Europace [Internet].

[48] El-Chami M.F., Garweg C., Clementy N. (2024). Leadless pacemakers at 5-year follow-up: the Micra transcatheter pacing system post-approval registry. Eur Heart J.

[49] Afzal M.R., Daoud E.G., Hussain S. (2019). Multicenter experience of feasibility and safety of leadless pacemakers across bioprosthetic and repaired tricuspid valves. JACC Clin Electrophysiol.

[50] Kerwin S.A., Mayotte M.J., Gornick C.C. (2015). Transcatheter pacemaker implantation in a patient with a bioprosthetic tricuspid valve. J Interv Cardiac Electrophysiol.

[51] Theis C., Huber C., Kaesemann P. (2020). Implantation of leadless pacing systems in patients early after tricuspid valve surgery: a feasible option. Pacing Clin Electrophysiol.

[52] Beurskens N.E.G., Tjong F.V.Y., Dasselaar K.J., Kuijt W.J., Wilde A.A.M., Knops R.E. (2019). Leadless pacemaker implantation after explantation of infected conventional pacemaker systems: a viable solution?. Heart Rhythm.

[53] del Corral M.P., Covas P., Tracy C., Mercader M., Solomon A. (2021). Percutaneous VDD leadless pacer implant post recent bioprosthetic tricuspid valve replacement for infective endocarditis. Pacing Clin Electrophysiol.

[54] Srinivasan N.T., Segal O.R. (2015). Biventricular pacing and coronary sinus ICD lead implantation in a patient with a mechanical tricuspid valve replacement. J Cardiol Cases.

[55] Lopez J.A. (2009). Total transvenous approach to pacing and defibrillation after Ebstein’s anomaly. Ann Thorac Surg.

[56] Schreiber C., Mehmanesch H., Kolb C., Schmitt C., Lange R. (2000). Modified implantation of a transvenous defibrillator in a patient after tricuspid valve replacement. Pacing Clin Electrophysiol.

[57] Biffi M., Bertini M., Ziacchi M., Boriani G. (2007). Transvenous cardioverter-defibrillator implantation in a patient with tricuspid mechanical prosthesis. J Cardiovasc Electrophysiol.

[58] Lopez J.A., Lufschanowski R. (2008). A novel approach to transvenous dual-chamber pacing lead placement and cardiac defibrillator implantation after tricuspid valve replacement. J Cardiovasc Electrophysiol.

[59] Knops R.E., Lloyd M.S., Roberts P.R. (2024). A modular communicative leadless pacing–defibrillator system. N Engl J Med.

[60] Friedman P., Murgatroyd F., Boersma L.V.A. (2022). Efficacy and safety of an extravascular implantable cardioverter–defibrillator. N Engl J Med.

[61] Cooper J.A., Smith T.W. (2009). How to implant a defibrillation coil in the azygous vein. Heart Rhythm.

[62] Hamid S., Arujna A., Khan S. (2009). Extraction of chronic pacemaker and defibrillator leads from the coronary sinus: laser infrequently used but required. Europace.

[63] Tonko J.B., Blauth C., Rosenthal E., Rinaldi C.A. (2021). Completely epicardial implantable cardioverter/defibrillator (ICD) and CRT-D systems: a case series and systematic literature review. Pacing Clin Electrophysiol.

[64] Carabelli A., Jabeur M., Jacon P. (2021). European experience with a first totally leadless cardiac resynchronization therapy pacemaker system. Europace.

[65] Okabe T., Hummel J.D., Bank A.J. (2022). Leadless left ventricular stimulation with WiSE-CRT System – Initial experience and results from phase I of SOLVE-CRT Study (nonrandomized, roll-in phase). Heart Rhythm.

[66] Webb J.G., Chuang A., Meier D. (2022). Transcatheter tricuspid valve replacement with the EVOQUE system: 1-year outcomes of a multicenter, first-in-human experience. JACC Cardiovasc Interv.

[67] Anderson J.H., McElhinney D.B., Aboulhosn J. (2020). Management and outcomes of transvenous pacing leads in patients undergoing transcatheter tricuspid valve replacement. JACC Cardiovasc Interv.

[68] Molina J.E., Roberts C.L., Benditt D.G. (2010). Long-term follow-up of permanent transvenous pacing systems preserved during tricuspid valve replacement. Ann Thorac Surg.

[69] Aris A., Callejo F., Cobiella J., Maestre M.L. (2004). Tricuspid valve replacement in the presence of an endocardial pacemaker electrode. J Heart Valve Dis.

[70] Miho T., Yoshikai M., Satoh H., Nakanishi H. (2017). Tricuspid valve replacement without removing transvenous pacemaker lead. Kyobu Geka.

[71] Steinberg C., Dvir D., Krahn A.D., Webb J. (2016). Feasibility of tricuspid valve-in-valve replacement in a patient with transvalvular pacemaker. HeartRhythm Case Rep.

[72] Paradis J.M., Bernier M., Houde C. (2015). Jailing of a pacemaker lead during tricuspid valve-in-valve implantation with an Edwards SAPIEN XT transcatheter heart valve. Can J Cardiol.

[73] Eleid M.F., Asirvatham S.J., Cabalka A.K. (2016). Transcatheter tricuspid valve-in-valve in patients with transvalvular device leads. Catheter Cardiovasc Interv.

[74] Kypta A., Blessberger H., Kammler J., Lambert T., Lichtenauer M., Steinwender C. (2016). Extraction of a trapped pacemaker lead in a pacemaker-dependent patient. J Cardiol Cases.

[75] Borleffs C.J.W., van Erven L., van Bommel R.J. (2009). Risk of failure of transvenous implantable cardioverter-defibrillator leads. Circ Arrhythm Electrophysiol Am Heart Assoc.

[76] Paz Rios LH., Alsaad A.A., Guerrero M., Metzl M.D. (2020). Tricuspid valve-in-valve jailing right ventricular lead is not free of risk. Catheter Cardiovasc Interv.

[77] Bhuta S., Colak S., Arif A.I., Afzal M.R. (2024). Snaring via a femoral approach to facilitate transvenous lead extraction of an infected right ventricular lead jailed by a bioprosthetic tricuspid valve. J Innov Card Rhythm Manag.

[78] Andreas M., Burri H., Praz F. (2024). Tricuspid valve disease and cardiac implantable electronic devices. Eur Heart J.

[79] Sidhu B.S., Gould J., Porter B. (2020). Completely leadless cardiac resynchronization defibrillator system. Clin Electrophysiol.

